# Supplementary material for: Serum Proteomic Profile of Asthmatic Patients after Six Months of Benralizumab and Mepolizumab Treatment
Source: Biomedicines. 2022 Mar 24;10(4):761. doi: 10.3390/biomedicines10040761 (PMC9027545; doi:10.3390/biomedicines10040761)
Supplement: Supplementary file 1 [file biomedicines-10-00761-s001.zip › Supplementary Table S2 Protein identification by mass spectrometry.pdf]

Spot N.	Protein description[a]	Uniprot ID/name[b]	AC	Gene name	MetaCore name	Mascot search results			
						Theorical pI-MW	Score	No. of matched peptides	Sequence coverage (%)
1	Alpha-2-macroglobulin	A2MG_HUMAN	P01023	A2M	A2M	6.03-164613	247	25/30	18
2	Alpha-2-macroglobulin	A2MG_HUMAN	P01023	A2M	A2M	6.03-164613	252	24/26	17
3	Plasminogen	PLMN_HUMAN	P00747	PLG	Plasminogen	7.04-93247	85	10/20	14
4	Plasminogen	PLMN_HUMAN	P00747	PLG	Plasminogen	7.04-93247	115	11/19	18
5	Plasminogen	PLMN_HUMAN	P00747	PLG	Plasminogen	7.04-93247	260	22/26	28
7	Complement factor B	CFAB_HUMAN	P00751	CFB	Factor B, Factor Ba, Factor Bb	6.67-86847	233	18/22	29
8	Complement factor B	CFAB_HUMAN	P00751	CFB	Factor B, Factor Ba, Factor Bb	6.67-86847	310	27/37	41
9	Complement factor B	CFAB_HUMAN	P00751	CFB	Factor B, Factor Ba, Factor Bb	6.67-86847	266	22/28	3
10	Serotransferrin	TRFE_HUMAN	P02787	TF	Trasferrin, Holotrasferrin	6.81-79294	96	9/12	12
11	Serotransferrin	TRFE_HUMAN	P02787	TF	Trasferrin, Holotrasferrin	6.81-79294	411	31/35	43
13	Serotransferrin	TRFE_HUMAN	P02787	TF	Trasferrin, Holotrasferrin	6.81-79294	402	37/55	47
14	Albumin	ALBU_HUMAN	P02768	ALB	Albumin	5.92-71317	106	5/9	16
15	Albumin	ALBU_HUMAN	P02768	ALB	Albumin	5.92-71317	252	23/36	34
16	Albumin	ALBU_HUMAN	P02768	ALB	Albumin	5.92-71317	92	8/13	13
18	Complement factor B	CFAB_HUMAN	P00751	CFB	Factor B, Factor Ba, Factor Bb	6.67-86847	104	11/20	17
19	Albumin	ALBU_HUMAN	P02768	ALB	Albumin	5.92-71317	345	29/37	43
20	Albumin	ALBU_HUMAN	P02768	ALB	Albumin	5.92-71317	71	5/6	9
21	Albumin	ALBU_HUMAN	P02768	ALB	Albumin	5.92-71317	297	25/33	41
22	Leucine-rich alpha-2-glycoprotein	A2GL_HUMAN	P02750	LRG1	LRG	6.45-38382	149	9/11	27
24	Albumin	ALBU_HUMAN	P02768	ALB	Albumin	5.92-71317	347	27/32	42
27	Albumin	ALBU_HUMAN	P02768	ALB	Albumin	5.92-71317	253	20/24	26
28	Albumin Fragment N	ALBU_HUMAN	P02768	ALB	Albumin	5.92-71317	112	10/15	15
29	Albumin Fragment N	ALBU_HUMAN	P02768	ALB	Albumin	5.92-71317	104	10/16	16
30	Immunoglobulin lambda constant 2	IGLC2_HUMAN	P0DOY2	IGLC2	LAC2	6.92-11458	51	3/10	46
34	Apolipoprotein C-III	APOC3_HUMAN	P02656	APOC3	HDL, APOC3	5.23-10845	71	4/10	3
35	Apolipoprotein C-III	APOC3_HUMAN	P02656	APOC3	HDL, APOC3	5.23-10845	73	4/10	45

37	Plasminogen	PLMN_HUMAN	P00747	PLG	Plasminogen	7.04-93247	191	17/25	26
38	Plasminogen	PLMN_HUMAN	P00747	PLG	Plasminogen	7.04-93247	301	25/32	35
40	Albumin	ALBU_HUMAN	P02768	ALB	Albumin	5.92-71317	326	24/25	35
41	Alpha-2-macroglobulin	A2MG_HUMAN	P01023	A2M	A2M	6.03-164613	325	29/29	24
42	Plasminogen	PLMN_HUMAN	P00747	PLG	Plasminogen	7.04-93247	104	11/22	2
43	Complement factor B	CFAB_HUMAN	P00751	CFB	Factor B, Factor Ba, Factor Bb	6.67-86847	83	7/11	13
44	Complement factor B	CFAB_HUMAN	P00751	CFB	Factor B, Factor Ba, Factor Bb	6.67-86847	351	27/33	44
45	Gelsolin	GELS_HUMAN	P06396	GSN	Gelsolin	5.90-86043	286	24/33	4
46	Albumin	ALBU_HUMAN	P02768	ALB	Albumin	5.92-71317	364	28/34	5
48	Albumin	ALBU_HUMAN	P02768	ALB	Albumin	5.92-71317	230	17/20	28
49	Albumin	ALBU_HUMAN	P02768	ALB	Albumin	5.92-71317	317	25/32	44
51	Albumin Fragment C	ALBU_HUMAN	P02768	ALB	Albumin	5.92-71317	253	22/31	37
52	Albumin	ALBU_HUMAN	P02768	ALB	Albumin	5.92-71317	166	11/12	18
53	Albumin	ALBU_HUMAN	P02768	ALB	Albumin	5.92-71317	246	17/18	26
55	Transthyretin	TTHY_HUMAN	P02766	TTR	Transthyretin	5.52-15991	184	5/13	69
56	Apolipoprotein C-II	APOC2_HUMAN	P02655	APOC2	HDL, APOC2	4.72-11277	88	10/20	47
57	Ceruloplasmin	CERU_HUMAN	P00450	CP	Ceruloplasmin	5.44-122983	111	11/16	14
61	Alpha-1-antichymotrypsin +	AACT_HUMAN	P01011	SERPINA3	SERPINA3 (ACT)	5.33-47792	182	19/35	34
	Vitronectin	VTNC_HUMAN	P04004	VTN	Vitronectin	5.55-55069	82	10/35	16
64	Albumin	ALBU_HUMAN	P02768	ALB	Albumin	5.92-71317	82	7/10	10
65	Albumin	ALBU_HUMAN	P02768	ALB	Albumin	5.92-71317	84	8/13	10
67	Albumin Fragment C	ALBU_HUMAN	P02768	ALB	Albumin	5.92-71317	184	15/21	28
69	Immunoglobulin lambda constant 2	IGLC2_HUMAN	P0DOY2	IGLC2	LAC2	6.92-11458	129	7/15	66
70	Ceruloplasmin	CERU_HUMAN	P00450	CP	Ceruloplasmin	5.44-122983	227	21/26	23
71	Alpha-1-antichymotrypsin	AACT_HUMAN	P01011	SERPINA3	SERPINA3 (ACT)	5.33-47792	116	9/15	25
73	Apolipoprotein A-I	APOA1_HUMAN	P02647	APOA1	HDL, Apoa1	5.56-30759	202	14/20	4
74	Alpha-1-antichymotrypsin	AACT_HUMAN	P01011	SERPINA3	SERPINA3 (ACT)	5.33-47792	192	15/23	35
75	Albumin	ALBU_HUMAN	P02768	ALB	Albumin	5.92-71317	115	10/15	15
76	Albumin Fragment C	ALBU_HUMAN	P02768	ALB	Albumin	5.92-71317	268	20/24	31
78	Transthyretin	TTHY_HUMAN	P02766	TTR	Transthyretin	5.52-15991	94	5/10	52
79	Apolipoprotein A-I	APOA1_HUMAN	P02647	APOA1	HDL, Apoa1	5.56-30759	264	21/30	55
81	Immunoglobulin J chain	IGJ_HUMAN	P01591	JCHAIN	IgJ	5.12-18543	82	5/9	29
82	Transthyretin	TTHY_HUMAN	P02766	TTR	Transthyretin	5.52-15991	104	5/7	44

Supplementary Table S2: Protein identification by mass spectrometry, showing spot numbers corresponding to Figure 1 and Table S-1, protein names, UniProt names, accession numbers and gene names, MetaCore names, theoretical p*I* and MW and Mascot Search Results (Score, Matched peptides, Coverage%).
