# Supplementary material for: Serum Proteomic Profile of Asthmatic Patients after Six Months of Benralizumab and Mepolizumab Treatment
Source: Biomedicines. 2022 Mar 24;10(4):761. doi: 10.3390/biomedicines10040761 (PMC9027545; doi:10.3390/biomedicines10040761)
Supplement: Supplementary file 1 [file biomedicines-10-00761-s001.zip › Supplementary Table S1 Statistical analysis of protein profiles.pdf]

Spot n°	Protein name	Kruskal-Wallis and Dunn'test		Mean ± SD						Ratio												
		p-value	adj. p-value	CTRL	T0	T1M	T1B	T6M	T6B	CTRLvsT0	CTRL vsT1M	CTRL vs T1B	CTRL vs T6M	CTRL vs T6B	T0 vs T1M	T0 vs T1B	T0 vs T6M	T0 vs T6B	T1M vs T6M	T1M vs T1B	T1B vs T6B	T6M vs T6B
1	Alpha-2-macroglobulin	1,01E-03	5,02E-03	0,02 ± 0,02	0,11 ± 0,04	0,14 ± 0,09	0,1 ± 0,06	0,05 ± 0,04	0,08 ± 0,02	**0,113**	**0,083**	**0,128**	**0,235**	**0,145**	0,735	1,138	**2,092**	**0,440**	**2,847**	1,549	1,132	0,616
2	Alpha-2-macroglobulin	8,45E-03	2,46E-02	0,01 ± 0,02	0,08 ± 0,03	0,12 ± 0,07	0,08 ± 0,05	0,07 ± 0,03	0,06 ± 0,04	**0,122**	**0,084**	**0,124**	**0,156**	**0,181**	0,685	1,011	1,278	0,572	1,866	1,477	1,464	1,159
3	Plasminogen	2,72E-04	1,96E-03	0,01 ± 0,01	0,02 ± 0,02	0,02 ± 0,01	0,03 ± 0,01	0,02 ± 0,01	0,03 ± 0,01	**0,142**	**0,211**	**0,095**	**0,135**	**0,100**	1,484	0,667	0,951	**0,437**	0,640	**0,450**	1,054	0,740
4	Plasminogen	8,72E-04	1,74E-02	0,01 ± 0,01	0,03 ± 0,02	0,05 ± 0,04	0,04 ± 0,02	0,03 ± 0,01	0,04 ± 0,02	**0,215**	**0,159**	**0,183**	**0,260**	**0,197**	0,740	0,853	1,209	0,556	1,634	1,153	1,073	0,757
5	Plasminogen	5,54E-03	1,81E-02	0,02 ± 0,01	0,04 ± 0,02	0,04 ± 0,03	0,03 ± 0,01	0,03 ± 0,01	0,03 ± 0,01	**0,321**	**0,264**	**0,385**	**0,497**	**0,376**	0,823	1,199	1,549	0,730	1,883	1,457	0,978	0,757
6		1,09E-04	1,05E-03	0,01 ± 0,01	0,02 ± 0,02	0,02 ± 0,01	0,03 ± 0,01	0,02 ± 0,01	0,02 ± 0,01	**0,433**	**0,456**	**0,307**	0,601	**0,455**	1,053	0,708	1,388	0,598	1,318	0,672	1,483	0,757
7	Complement factor B	6,17E-04	3,61E-03	0,04 ± 0,03	0,09 ± 0,03	0,13 ± 0,05	0,09 ± 0,02	0,11 ± 0,05	0,14 ± 0,03	**0,420**	**0,307**	**0,448**	**0,354**	**0,282**	0,731	1,067	0,843	**0,163**	1,153	1,459	0,630	0,797
8	Complement factor B	1,66E-04	1,37E-03	0,07 ± 0,04	0,12 ± 0,02	0,15 ± 0,03	0,13 ± 0,02	0,12 ± 0,03	0,15 ± 0,04	0,517	**0,442**	**0,484**	0,534	**0,422**	0,854	0,937	1,032	**0,129**	1,209	1,097	0,872	0,791
9	Complement factor B	4,29E-03	1,46E-02	0,02 ± 0,02	0,06 ± 0,03	0,07 ± 0,04	0,06 ± 0,02	0,04 ± 0,02	0,06 ± 0,03	**0,371**	**0,295**	**0,383**	0,605	**0,356**	0,794	1,030	1,629	0,537	**2,052**	1,297	0,931	0,588
10	Serotransferrin	2,16E-03	8,71E-03	0,02 ± 0,01	0,04 ± 0,02	0,03 ± 0,01	0,02 ± 0,02	0,03 ± 0,01	0,03 ± 0,01	**0,404**	0,544	0,972	0,504	0,501	1,346	**2,404**	1,245	**0,383**	0,925	1,785	0,516	0,996
11	Serotransferrin	9,82E-05	9,82E-04	0,04 ± 0,05	0,12 ± 0,04	0,1 ± 0,02	0,1 ± 0,02	0,09 ± 0,03	0,1 ± 0,02	**0,293**	**0,366**	**0,367**	**0,374**	**0,339**	1,249	1,253	1,276	**0,384**	1,022	1,003	0,924	0,907
12		9,74E-04	4,89E-03	0	0,06 ± 0,04	0,07 ± 0,04	0,04 ± 0,02	0,05 ± 0,02	0,05 ± 0,04	**0**	**0**	**0**	**0**	**0**	0,890	1,625	1,238	0,771	1,391	1,826	0,887	1,164
13	Serotransferrin	1,46E-04	1,25E-03	0,19 ± 0,21	0,32 ± 0,1	0,36 ± 0,15	0,4 ± 0,09	0,44 ± 0,05	0,33 ± 0,1	0,591	0,512	**0,471**	**0,426**	0,567	0,867	0,796	0,720	**0,297**	0,831	0,919	1,204	1,330
14	Albumin	3,69E-04	2,49E-03	0,06 ± 0,03	0,18 ± 0,07	0,2 ± 0,04	0,16 ± 0,07	0,12 ± 0,02	0,21 ± 0,1	**0,304**	**0,272**	**0,345**	**0,449**	**0,253**	0,893	1,134	1,476	**0,314**	1,654	1,270	0,732	0,562
15	Albumin	1,61E-05	2,86E-04	0,09 ± 0,05	0,22 ± 0,07	0,27 ± 0,03	0,23 ± 0,1	0,16 ± 0,03	0,21 ± 0,02	**0,405**	**0,337**	**0,394**	0,585	**0,437**	0,833	0,974	1,445	**0,340**	1,735	1,169	1,108	0,747
16	Albumin	1,19E-03	5,62E-03	0,03 ± 0,03	0,08 ± 0,04	0,08 ± 0,06	0,13 ± 0,03	0,07 ± 0,02	0,08 ± 0,02	**0,383**	**0,380**	**0,238**	**0,424**	**0,382**	0,994	0,621	1,107	**0,417**	1,114	0,625	1,606	0,901
17		1,05E-02	2,92E-02	0,01 ± 0,01	0,02 ± 0,02	0,01 ± 0,01	0,01 ± 0,01	0,04 ± 0,04	0,01 ± 0,01	**0,041**	**0,090**	**0,175**	**0,023**	**0,129**	**2,219**	**4,308**	0,571	1,808	**0,257**	1,941	0,738	**5,567**
18	Complement factor B	2,93E-05	4,32E-04	0,01 ± 0,01	0,04 ± 0,03	0,04 ± 0,02	0,02 ± 0,01	0,01 ± 0,01	0,01 ± 0,01	**0,060**	**0,057**	**0,163**	**0,240**	**0,235**	0,954	**2,721**	**4,014**	**2,500**	**4,209**	**2,853**	1,447	0,981
19	Albumin	1,38E-03	6,21E-03	0,11 ± 0,05	0,21 ± 0,07	0,23 ± 0,04	0,23 ± 0,1	0,17 ± 0,02	0,16 ± 0,01	**0,495**	**0,443**	**0,448**	0,628	0,651	0,895	0,904	1,268	**0,399**	1,416	1,010	1,453	1,036
20	Albumin	2,39E-04	1,78E-03	0,04 ± 0,03	0,09 ± 0,04	0,08 ± 0,05	0,12 ± 0,03	0,09 ± 0,02	0,08 ± 0,01	**0,364**	**0,429**	**0,273**	**0,375**	**0,393**	1,179	0,750	1,033	**0,424**	0,876	0,636	1,443	1,048
21	Albumin	4,57E-04	2,94E-03	0,06 ± 0,03	0,1 ± 0,03	0,11 ± 0,02	0,1 ± 0,04	0,1 ± 0,02	0,12 ± 0,04	0,561	**0,476**	0,558	0,538	**0,435**	0,848	0,994	0,959	**0,250**	1,130	1,172	0,780	0,809
22	Leucine-rich alpha-2-glycoprotein	2,16E-03	8,71E-03	0,05 ± 0,02	0,02 ± 0,02	0,02 ± 0,01	0,02 ± 0,01	0,03 ± 0,02	0,04 ± 0,03	**2,776**	**2,768**	**2,280**	1,556	1,159	0,997	0,821	0,561	**0,396**	0,562	0,824	0,508	0,745
23		4,22E-03	1,44E-02	0,02 ± 0,01	0,06 ± 0,04	0,07 ± 0,04	0,04 ± 0,03	0,06 ± 0,04	0,04 ± 0,03	**0,303**	**0,223**	**0,468**	**0,297**	**0,411**	0,735	1,544	0,977	0,806	1,330	**2,101**	0,877	1,385
24	Albumin	1,41E-08	2,19E-06	0,06 ± 0,03	0,12 ± 0,05	0,13 ± 0,03	0,08 ± 0,02	0,01 ± 0,02	0,07 ± 0,02	**0,497**	**0,464**	0,761	**12,823**	0,861	0,934	1,533	**25,827**	0,608	**27,657**	1,642	1,131	**0,067**
25		2,23E-04	1,69E-03	0,03 ± 0,02	0,05 ± 0,01	0,05 ± 0,02	0,05 ± 0,02	0,03 ± 0,01	0,03 ± 0,01	**0,422**	**0,489**	**0,441**	0,818	0,795	1,160	1,046	1,940	**0,376**	1,672	0,902	1,802	0,971
26		6,15E-06	1,37E-04	0,03 ± 0,02	0,06 ± 0,03	0,07 ± 0,02	0,05 ± 0,01	0,04 ± 0,01	0,05 ± 0,01	0,517	**0,468**	0,612	0,750	0,679	0,905	1,183	1,451	0,510	1,603	1,307	1,110	0,906
27	Albumin	3,47E-05	4,96E-04	0,05 ± 0,02	0,1 ± 0,05	0,11 ± 0,03	0,08 ± 0,02	0,07 ± 0,01	0,07 ± 0,02	**0,497**	**0,472**	0,629	0,783	0,731	0,949	1,266	1,576	0,644	1,660	1,333	1,162	0,933
28	Albumin Fragment N	1,48E-05	2,73E-04	0,05 ± 0,02	0,09 ± 0,04	0,11 ± 0,03	0,08 ± 0,03	0,07 ± 0,01	0,07 ± 0,01	**0,492**	**0,425**	0,592	0,727	0,712	0,863	1,204	1,478	0,581	1,712	1,395	1,202	0,979
29	Albumin Fragment N	1,14E-04	1,07E-03	0,03 ± 0,02	0,06 ± 0,02	0,06 ± 0,02	0,05 ± 0,02	0,04 ± 0,01	0,04 ± 0,01	0,521	**0,487**	0,679	0,827	0,810	0,934	1,303	1,587	0,527	1,700	1,396	1,192	0,979
30	Immunoglobulin lambda constant 2	1,17E-02	3,17E-02	0,08 ± 0,04	0,2 ± 0,07	0,21 ± 0,07	0,19 ± 0,13	0,13 ± 0,03	0,14 ± 0,02	**0,364**	**0,340**	**0,376**	0,553	0,537	0,934	1,034	1,521	0,525	1,628	1,107	1,426	0,970
31		4,27E-06	1,10E-04	0,03 ± 0,02	0,11 ± 0,03	0,1 ± 0,04	0,11 ± 0,03	0,06 ± 0,02	0,06 ± 0,03	**0,257**	**0,288**	**0,256**	0,532	**0,499**	1,117	0,995	**2,066**	0,502	1,849	0,890	1,951	0,939
32		6,27E-03	1,98E-02	0,08 ± 0,06	0,18 ± 0,15	0,26 ± 0,1	0,14 ± 0,08	0,11 ± 0,02	0,21 ± 0,17	**0,402**	**0,283**	0,530	0,673	**0,351**	0,704	1,319	1,674	0,687	**2,380**	1,874	0,662	0,522

33		9,50E-03	2,72E-02	0,02 ± 0,02	0,05 ± 0,04	0,06 ± 0,03	0,03 ± 0,02	0,03 ± 0,01	0,06 ± 0,05	**0,365**	**0,343**	0,603	0,660	**0,290**	0,939	1,650	1,807	0,632	1,924	1,757	**0,481**	**0,439**
34	Apolipoprotein C-III	2,51E-06	8,09E-05	0,1 ± 0,04	0,05 ± 0,03	0,02 ± 0,02	0,06 ± 0,05	0,08 ± 0,03	0,14 ± 0,02	**2,315**	**5,572**	1,834	1,301	0,666	**2,407**	0,792	0,562	**0,172**	**0,233**	**0,329**	**0,363**	0,512
35	Apolipoprotein C-III	1,60E-05	2,86E-04	0,14 ± 0,08	0,05 ± 0,03	0,03 ± 0,02	0,05 ± 0,05	0,07 ± 0,02	0,14 ± 0,02	**3,470**	**5,785**	**3,438**	**2,209**	1,018	1,667	0,991	0,637	**0,203**	**0,382**	0,594	**0,296**	**0,461**
36		4,17E-05	5,61E-04	0,09 ± 0,04	0,05 ± 0,02	0,05 ± 0,02	0,04 ± 0,01	0,07 ± 0,02	0,08 ± 0,02	1,978	1,944	**2,088**	1,233	1,033	0,983	1,056	0,623	**0,189**	0,634	1,074	**0,495**	0,837
37	Plasminogen	2,28E-03	9,12E-03	0,02 ± 0,02	0,04 ± 0,02	0,03 ± 0,02	0,04 ± 0,02	0,03 ± 0,01	0,04 ± 0,01	**0,358**	**0,399**	**0,302**	**0,402**	**0,292**	1,115	0,844	1,125	0,521	1,009	0,757	0,967	0,725
38	Plasminogen	4,25E-02	1,53E-03	0,01 ± 0,01	0,04 ± 0,03	0,02 ± 0,02	0,03 ± 0,03	0,03 ± 0,03	0,04 ± 0,01	**0,174**	**0,301**	**0,218**	**0,224**	**0,171**	1,732	1,258	1,288	0,808	0,743	0,726	0,784	0,766
39		4,63E-05	6,06E-04	0,01 ± 0,01	0,02 ± 0,02	0,01 ± 0,01	0,03 ± 0,01	0,01 ± 0,01	0,02 ± 0,01	**0,041**	**0,112**	**0,031**	**0,076**	**0,062**	**2,746**	0,757	1,874	1,283	0,682	**0,276**	1,994	0,805
40	Albumin	1,20E-04	1,11E-03	0,03 ± 0,03	0,07 ± 0,02	0,08 ± 0,01	0,08 ± 0,03	0,05 ± 0,02	0,05 ± 0,01	**0,351**	**0,291**	**0,314**	**0,475**	0,557	0,829	0,895	1,355	**0,400**	1,634	1,079	1,775	1,172
41	Alpha-2-macroglobulin	1,96E-02	4,65E-02	0,04 ± 0,07	0,11 ± 0,07	0,19 ± 0,15	0,12 ± 0,08	0,04 ± 0,04	0,06 ± 0,03	**0,306**	**0,178**	**0,280**	1,046	0,648	0,581	0,917	**3,420**	1,191	**5,881**	1,578	**2,311**	0,620
42	Plasminogen	2,14E-02	4,96E-02	0,01 ± 0,01	0,03 ± 0,02	0,03 ± 0,02	0,02 ± 0,02	0,03 ± 0,02	0,04 ± 0,01	**0,437**	**0,402**	0,770	**0,338**	**0,270**	0,922	1,763	0,775	**0,453**	0,841	1,914	**0,350**	0,797
43	Complement factor B	4,73E-03	1,57E-02	0,02 ± 0,02	0,07 ± 0,04	0,08 ± 0,04	0,05 ± 0,02	0,06 ± 0,04	0,08 ± 0,02	**0,203**	**0,168**	**0,260**	**0,230**	**0,167**	0,829	1,282	1,132	**0,441**	1,364	1,546	0,643	0,728
44	Complement factor B	5,91E-03	1,90E-02	0,06 ± 0,03	0,1 ± 0,04	0,12 ± 0,04	0,13 ± 0,04	0,09 ± 0,02	0,13 ± 0,05	0,633	0,526	**0,456**	0,692	**0,483**	0,831	0,720	1,093	**0,291**	1,315	0,867	1,059	0,698
45	Gelsolin	4,82E-04	3,08E-03	0,05 ± 0,03	0,1 ± 0,05	0,15 ± 0,04	0,12 ± 0,05	0,1 ± 0,02	0,12 ± 0,03	**0,426**	**0,275**	**0,341**	**0,423**	**0,365**	0,645	0,800	0,993	**0,370**	1,539	1,240	1,071	0,863
46	Albumin	1,10E-02	3,02E-02	0,02 ± 0,02	0,05 ± 0,03	0,06 ± 0,02	0,07 ± 0,03	0,05 ± 0,01	0,05 ± 0,01	**0,370**	**0,327**	**0,267**	**0,400**	**0,402**	0,886	0,722	1,082	0,697	1,221	0,814	1,507	1,005
47		7,97E-04	4,36E-03	0,01 ± 0,01	0,03 ± 0,02	0,03 ± 0,02	0,02 ± 0,01	0,04 ± 0,01	0,03 ± 0,02	**0,090**	**0,106**	**0,125**	**0,078**	**0,079**	1,178	1,393	0,869	**0,439**	0,737	1,182	0,629	1,008
48	Albumin	2,11E-02	4,91E-02	0,05 ± 0,04	0,09 ± 0,03	0,11 ± 0,03	0,09 ± 0,03	0,13 ± 0,05	0,1 ± 0,05	0,536	**0,430**	0,540	**0,368**	0,508	0,803	1,007	0,687	**0,279**	0,856	1,254	0,941	1,379
49	Albumin	1,98E-02	4,68E-02	0,07 ± 0,05	0,11 ± 0,07	0,13 ± 0,06	0,11 ± 0,06	0,05 ± 0,03	0,04 ± 0,02	0,595	**0,481**	0,570	1,271	1,617	0,809	0,959	**2,137**	1,708	**2,641**	1,185	**2,835**	1,272
51	Albumin Fragment C	1,53E-02	3,87E-02	0,09 ± 0,04	0,15 ± 0,05	0,2 ± 0,05	0,16 ± 0,08	0,15 ± 0,03	0,15 ± 0,02	0,613	**0,453**	0,583	0,601	0,626	0,739	0,951	0,979	**0,321**	1,326	1,287	1,074	1,042
52	Albumin	1,98E-04	1,56E-03	0,03 ± 0,03	0,06 ± 0,03	0,05 ± 0,03	0,08 ± 0,03	0,08 ± 0,01	0,08 ± 0,02	**0,439**	**0,498**	**0,312**	**0,338**	**0,340**	1,134	0,710	0,771	**0,286**	0,680	0,626	1,090	1,003
53	Albumin	3,14E-04	2,21E-03	0,03 ± 0,03	0,04 ± 0,02	0,05 ± 0,01	0,04 ± 0,02	0,03 ± 0,02	0,04 ± 0,02	0,565	**0,488**	0,549	0,873	0,652	0,863	0,971	1,544	**0,358**	1,789	1,126	1,188	0,747
54		1,54E-02	3,89E-02	0,15 ± 0,08	0,26 ± 0,09	0,33 ± 0,14	0,15 ± 0,07	0,22 ± 0,08	0,23 ± 0,08	0,559	**0,440**	0,997	0,652	0,640	0,788	1,785	1,167	**0,371**	1,482	**2,267**	0,642	0,982
55	Transthyretin	2,79E-06	8,45E-05	0,08 ± 0,08	0,33 ± 0,11	0,4 ± 0,17	0,47 ± 0,05	0,25 ± 0,05	0,29 ± 0,12	**0,240**	**0,196**	**0,166**	**0,315**	**0,272**	0,815	0,690	1,314	**0,358**	1,611	0,847	1,643	0,863
56	Apolipoprotein C-II	2,56E-05	3,98E-04	0,05 ± 0,03	0,02 ± 0,02	0,01 ± 0,01	0,03 ± 0,01	0,05 ± 0,02	0,08 ± 0,02	**2,593**	**5,484**	1,984	1,214	0,687	**2,115**	0,765	**0,468**	**0,228**	**0,221**	**0,362**	**0,346**	0,566
57	Ceruloplasmin	9,05E-05	9,53E-04	0,02 ± 0,01	0,02 ± 0,01	0,01 ± 0,01	0,04 ± 0,02	0,04 ± 0,01	0,04 ± 0,01	1,279	1,612	**0,418**	**0,448**	**0,463**	1,260	**0,327**	**0,350**	**0,239**	**0,278**	**0,259**	1,108	1,035
58		1,14E-02	3,11E-02	0,02 ± 0,03	0,01 ± 0,01	0	0,01 ± 0,01	0,02 ± 0,01	0,04 ± 0,02	**3,215**	-	**2,245**	1,015	0,557	-	0,698	**0,316**	**0,249**	**0**	**0**	**0,248**	0,548
59		2,01E-03	8,26E-03	0,02 ± 0,02	0,01 ± 0,01	0,01 ± 0,01	0,01 ± 0,01	0	0	**2,115**	**2,935**	**8,017**	-	-	1,387	**3,790**	-	-	-	**2,732**	-	-
60		2,05E-06	7,22E-05	0	0,07 ± 0,02	0,08 ± 0,04	0,03 ± 0,02	0,06 ± 0,02	0,08 ± 0,03	**0**	**0**	**0**	**0**	**0**	0,900	**3,172**	1,146	**0,227**	1,273	**3,523**	**0,282**	0,782
61	Alpha-1-antichymotrypsin + vitronectin	6,18E-03	1,97E-02	0,06 ± 0,08	0,02 ± 0,03	0,04 ± 0,01	0,05 ± 0,01	0,09 ± 0,03	0	**3,742**	1,560	1,293	0,633	-	**0,417**	**0,346**	**0,169**	-	**0,406**	0,829	-	-
62		1,02E-04	1,01E-03	0,01 ± 0,01	0,02 ± 0,02	0,03 ± 0,01	0,01 ± 0,01	0,01 ± 0,01	0,01 ± 0,01	**0,305**	**0,266**	0,621	1,159	0,936	0,871	**2,037**	**3,801**	1,840	**4,362**	**2,338**	1,508	0,808
63		5,96E-07	3,09E-05	0	0,02 ± 0,02	0,03 ± 0,02	0	0	0	**0**	**0**	-	-	-	0,621	-	-	-	-	-	-	-
64	Albumin	3,01E-04	2,13E-03	0,09 ± 0,08	0,21 ± 0,08	0,21 ± 0,05	0,16 ± 0,05	0,09 ± 0,02	0,06 ± 0,02	**0,421**	**0,427**	0,570	0,993	1,556	1,014	1,353	**2,358**	1,263	**2,326**	1,334	**2,731**	1,566
65	Albumin	3,77E-08	4,51E-06	0,03 ± 0,03	0,07 ± 0,03	0	0,06 ± 0,02	0	0	**0,416**	-	**0,465**	-	-	-	1,116	-	-	-	**0**	-	-
66		1,83E-06	6,75E-05	0,08 ± 0,04	0,11 ± 0,04	0,12 ± 0,03	0,07 ± 0,02	0,05 ± 0,05	0,02 ± 0,03	0,723	0,662	1,138	1,716	**6,738**	0,916	1,574	**2,373**	**2,788**	**2,592**	1,719	**5,922**	**3,927**
67	Albumin Fragment C	8,23E-04	4,42E-03	0,03 ± 0,02	0,07 ± 0,03	0,09 ± 0,03	0,08 ± 0,04	0,02 ± 0,02	0,04 ± 0,02	**0,401**	**0,333**	**0,378**	1,427	0,881	0,830	0,943	**3,559**	0,979	**4,285**	1,135	**2,329**	0,617
68		8,78E-05	9,40E-04	0,02 ± 0,01	0,05 ± 0,02	0,04 ± 0,02	0,04 ± 0,02	0,02 ± 0,01	0,03 ± 0,01	**0,346**	**0,394**	**0,407**	0,829	0,688	1,138	1,176	**2,396**	0,724	**2,106**	1,034	1,691	0,831

69	Immunoglobulin lambda constant 2	4,94E-04	3,12E-03	0,2 ± 0,07	0,36 ± 0,16	0,45 ± 0,07	0,34 ± 0,18	0,18 ± 0,07	0,17 ± 0,03	0,559	**0,445**	0,593	1,151	1,238	0,797	1,061	**2,059**	0,939	**2,583**	1,332	**2,088**	1,076
70	Ceruloplasmin	1,19E-03	5,62E-03	0,03 ± 0,02	0,03 ± 0,02	0,03 ± 0,02	0,06 ± 0,03	0,05 ± 0,02	0,05 ± 0,02	1,119	1,231	**0,489**	0,522	0,554	1,100	**0,437**	**0,466**	**0,284**	**0,424**	**0,397**	1,134	1,062
71	Alpha-1-antichymotrypsin	1,92E-04	3,50E-03	0,04 ± 0,05	0,02 ± 0,03	0,02 ± 0,02	0,03 ± 0,01	0,05 ± 0,02	0,04 ± 0,02	**2,156**	**3,109**	1,477	0,874	0,954	1,442	0,685	**0,405**	0,504	**0,281**	**0,475**	0,646	1,091
72		6,41E-11	2,99E-08	0	0,01 ± 0,01	0,01 ± 0,01	0,01 ± 0,01	0,03 ± 0,01	0,04 ± 0,02	**0**	**0**	**0**	**0**	**0**	**2,143**	1,913	**0,122**	**0,113**	**0,057**	0,893	**0,050**	0,788
73	Apolipoprotein A-I	1,71E-02	4,17E-02	0,07 ± 0,05	0,03 ± 0,01	0,03 ± 0,02	0,02 ± 0,01	0,06 ± 0,04	0,07 ± 0,03	**2,867**	**2,770**	**4,454**	1,027	0,960	0,966	1,553	**0,358**	**0,107**	**0,371**	1,608	**0,215**	0,934
74	Alpha-1-antichymotrypsin	7,50E-03	2,24E-02	0,11 ± 0,09	0,05 ± 0,05	0,1 ± 0,02	0,05 ± 0,03	0,08 ± 0,03	0,17 ± 0,06	**2,303**	1,149	**2,367**	1,327	0,632	**0,499**	1,028	0,577	**0,281**	1,155	**2,060**	**0,267**	**0,476**
75	Albumin	3,93E-04	6,11E-03	0,03 ± 0,02	0,03 ± 0,01	0,03 ± 0,01	0,02 ± 0,01	0,04 ± 0,02	0,04 ± 0,01	0,817	0,769	1,149	0,599	0,516	0,941	1,406	0,733	**0,170**	0,779	1,494	**0,449**	0,861
76	Albumin Fragment C	1,24E-14	8,68E-12	0,01 ± 0,01	0,02 ± 0,01	0	0,01 ± 0,01	0,07 ± 0,02	0,06 ± 0,02	**0,131**	**-**	**0,160**	**0,021**	**0,024**	**-**	1,221	**0,163**	**0,104**	**0**	**0**	**0,148**	1,102
77		1,80E-03	2,65E-02	0,02 ± 0,03	0,02 ± 0,01	0,03 ± 0,01	0,02 ± 0,01	0,03 ± 0,02	0,04 ± 0,02	0,880	0,505	0,933	0,577	**0,338**	0,574	1,060	0,656	**0,199**	1,144	1,848	**0,362**	0,585
78	Transthyretin	6,01E-05	7,14E-04	0	0,02 ± 0,02	0,02 ± 0,01	0,01 ± 0,01	0,04 ± 0,02	0,05 ± 0,02	**0**	**0**	**0**	**0**	**0**	0,865	1,626	**0,488**	**0,376**	0,565	1,880	**0,201**	0,668
79	Apolipoprotein A-I	1,53E-03	6,68E-03	0,19 ± 0,09	0,15 ± 0,04	0,14 ± 0,04	0,12 ± 0,01	0,24 ± 0,05	0,25 ± 0,07	1,273	1,386	1,554	0,787	0,760	1,089	1,221	0,619	**0,143**	0,568	1,121	**0,489**	0,965
80		4,57E-04	2,94E-03	0,02 ± 0,02	0,02 ± 0,01	0,01 ± 0,01	0,01 ± 0,01	0,03 ± 0,01	0,03 ± 0,01	1,038	1,487	**4,145**	0,575	0,648	1,433	**3,994**	0,554	**0,371**	**0,387**	**2,788**	**0,156**	1,127
81	Immunoglobulin J chain	2,91E-03	3,57E-02	0,01 ± 0,01	0,01 ± 0,01	0,01 ± 0,01	0,01 ± 0,01	0,02 ± 0,01	0,02 ± 0,01	**2,205**	1,639	**2,715**	0,724	0,596	0,743	1,231	**0,328**	**0,365**	**0,442**	1,657	**0,220**	0,823
82	Transthyretin	4,31E-06	1,10E-04	0,13 ± 0,06	0,09 ± 0,03	0,08 ± 0,02	0,08 ± 0,02	0,16 ± 0,04	0,21 ± 0,03	1,414	1,569	1,607	0,795	0,605	1,109	1,137	0,562	**0,139**	0,507	1,025	**0,376**	0,760

Supplementary Table S1: Kruskal Wallis and Dunn's test statistical analysis of protein profiles, showing spot numbers, protein names, means and standard deviations (%V) and ratios of the mean %V, of CTRL, T0, T1M, T1B, T6M and T6B serum samples. Numbers in bold font indicate a statistically valid ratio.
