# Supplementary material for: Serum Proteomic Profile of Asthmatic Patients after Six Months of Benralizumab and Mepolizumab Treatment
Source: Biomedicines. 2022 Mar 24;10(4):761. doi: 10.3390/biomedicines10040761 (PMC9027545; doi:10.3390/biomedicines10040761)
Supplement: Supplementary file 1 [file biomedicines-10-00761-s001.zip › Supplementary Table S3 MetaCore Analysis.pdf]

Benralizumab Timepoints

#	Network	GO processes	Total nodes	Seed nodes	p-Value	zScore	gScore
1	Angiostatin, Plasmin, PPAR-gamma, PLAU (UPA), RelA (p65 NF-kB subunit)	response to nutrient (58.8%; 1.194e-13), fibrinolysis (29.4%; 5.311e-12), response to nutrient levels (64.7%; 1.028e-11), response to extracellular stimulus (64.7%; 1.781e-11), negative regulation of blood coagulation (35.3%; 2.878e-11)	21	9	5,13E-35	275,81	275,81
2	Albumin, NANOG, SOX2, GATA-4, c-Myc	gastrulation (44.0%; 2.684e-15), formation of primary germ layer (40.0%; 3.406e-15), positive regulation of cellular biosynthetic process (76.0%; 1.048e-13), positive regulation of biosynthetic process (76.0%; 1.527e-13), embryo development (64.0%; 8.385e-13)	25	9	6,23E-34	246,69	246,69
3	APOA1, SR-BI, S1P3 receptor, S1P1 receptor, Pre beta-1 HDL lipids extracellular region	high-density lipoprotein particle remodeling (44.8%; 2.076e-30), plasma lipoprotein particle remodeling (48.3%; 3.007e-30), protein-lipid complex remodeling (48.3%; 3.007e-30), protein-containing complex remodeling (48.3%; 1.133e-29), organic hydroxy compound transport (62.1%; 3.698e-29)	41	9	1,6E-32	211,53	211,53
4	Transthyretin, E2F1, TCF7L2 (TCF4), Myelin basic protein, 4E-BP2	response to endogenous stimulus (75.0%; 3.563e-21), regulation of multicellular organismal process (82.5%; 1.279e-19), response to hormone (62.5%; 2.295e-19), response to nutrient levels (52.5%; 1.386e-18), response to extracellular stimulus (52.5%; 3.908e-18)	48	9	4,16E-31	179,9	179,9
5	SERPINA3 (ACT), Oncostatin M, BMP7, IL-6, Thrombopoietin	cellular response to cytokine stimulus (70.0%; 2.829e-30), cytokine-mediated signaling pathway (60.0%; 1.134e-29), response to cytokine (70.0%; 1.804e-28), cellular response to organic substance (80.0%; 1.221e-23), cellular response to interleukin-6 (26.0%; 4.311e-22)	52	8	6,57E-27	153,5	153,5
6	Ceruloplasmin, IL-1 beta, AP-1, c-Jun, TAK1(MAP3K7)	activation of MAPK activity (45.0%; 1.508e-13), positive regulation of MAP kinase activity (45.0%; 5.515e-12), MAPK cascade (50.0%; 8.857e-12), positive regulation of MAPK cascade (55.0%; 1.108e-11), activation of protein kinase activity (45.0%; 5.677e-11)	20	7	2,46E-26	214,51	214,51

Mepolizumab Timepoints

Network	GO processes	Total nodes	Seed nodes	p-Value	zScore	gScore
Angiostatin, Plasmin, PPAR-gamma, PLAU (UPA), RelA (p65 NF-kB subunit)	response to nutrient (58.8%; 1.194e-13), fibrinolysis (29.4%; 5.311e-12), response to nutrient levels (64.7%; 1.028e-11), response to extracellular stimulus (64.7%; 1.781e-11), negative regulation of blood coagulation (35.3%; 2.878e-11)	21	9	5,280E-36	302,14	302,14
Albumin, NANOG, SOX2, GATA-4, KLF4	gastrulation (45.8%; 1.519e-15), formation of primary germ layer (41.7%; 2.059e-15), embryo development (66.7%; 3.242e-13), response to endogenous stimulus (75.0%; 3.347e-13), tissue morphogenesis (54.2%; 5.284e-13)	24	8	1,120E-30	245,16	245,16
APOA1, SR-BI, S1P3 receptor, S1P1 receptor, Pre beta-1 HDL lipids extracellular region	plasma lipoprotein particle remodeling (48.1%; 4.179e-28), protein-lipid complex remodeling (48.1%; 4.179e-28), high-density lipoprotein particle remodeling (44.4%; 5.027e-28), organic hydroxy compound transport (63.0%; 9.344e-28), protein-containing complex remodeling (48.1%; 1.414e-27)	39	7	2,160E-25	185,77	185,77
SERPINA3 (ACT), Oncostatin M, BMP7, IL-6, Thrombopoietin	cellular response to cytokine stimulus (71.4%; 9.055e-31), cytokine-mediated signaling pathway (61.2%; 4.712e-30), response to cytokine (71.4%; 5.827e-29), cellular response to organic substance (81.6%; 2.880e-24), regulation of cell population proliferation (71.4%; 2.241e-23)	51	7	6,400E-24	148,6	148,6
Ceruloplasmin, IL-1 beta, AP-1, c-Jun, TAK1(MAP3K7)	activation of MAPK activity (47.4%; 8.365e-14), positive regulation of MAP kinase activity (47.4%; 3.074e-12), MAPK cascade (52.6%; 4.525e-12), positive regulation of MAPK cascade (57.9%; 5.147e-12), response to stress (94.7%; 2.612e-11)	19	6	6,540E-23	206,65	206,65
Pre beta-1 HDL lipids extracellular region, SR-BI, S1P1 receptor, S1P3 receptor, APOA1	protein-lipid complex remodeling (85.7%; 1.016e-30), plasma lipoprotein particle remodeling (85.7%; 1.016e-30), protein-containing complex remodeling (85.7%; 3.102e-30), high-density lipoprotein particle remodeling (78.6%; 9.674e-30), plasma lipoprotein particle organization (85.7%; 8.638e-29)	23	5	5,040E-19	182,06	182,06

7	SAR1A, Sec23, PKC, MTP, APOA4	regulation of lipid localization (53.1%; 1.031e-24), plasma lipoprotein particle remodeling (37.5%; 2.442e-24), protein-lipid complex remodeling (37.5%; 2.442e-24), lipoprotein transport (34.4%; 4.735e-24), lipoprotein localization (34.4%; 6.590e-24)	56	7	1,02E-23	146,28	146,28
8	Pre beta-1 HDL lipids extracellular region, SR-BI, S1P1 receptor, S1P3 receptor, APOA1	protein-lipid complex remodeling (85.7%; 1.016e-30), plasma lipoprotein particle remodeling (85.7%; 1.016e-30), protein-containing complex remodeling (85.7%; 3.102e-30), high-density lipoprotein particle remodeling (78.6%; 9.674e-30), plasma lipoprotein particle organization (85.7%; 8.638e-29)	23	5	1,44E-18	166,19	166,19
9	APOC3, LPL, Biglycan proteoglycan, Decorin proteoglycan, Versican proteoglycan	chylomicron remodeling (53.3%; 2.792e-20), triglyceride-rich lipoprotein particle remodeling (53.3%; 6.243e-20), protein-lipid complex remodeling (53.3%; 4.357e-18), plasma lipoprotein particle remodeling (53.3%; 4.357e-18), regulation of plasma lipoprotein particle levels (60.0%; 5.433e-18)	21	4	2,72E-14	122,57	122,57
10	S1P1 receptor, S1P3 receptor, APOA1, Pre beta-1 HDL, PERM	protein-lipid complex remodeling (81.8%; 7.797e-23), plasma lipoprotein particle remodeling (81.8%; 7.797e-23), protein-containing complex remodeling (81.8%; 1.758e-22), plasma lipoprotein particle organization (81.8%; 2.006e-21), high-density lipoprotein particle remodeling (72.7%; 2.636e-21)	14	3	2,78E-11	118,68	118,68
11	Pre beta-1 HDL lipids extracellular region, Pre beta-1 HDL lipids + Cholesterol + Phospholipid = HDL nascent lipids, HDL nascent lipids extracellular region, Cholesterol extracellular region	cholesterol import (100.0%; 2.069e-10), sterol import (100.0%; 2.069e-10), negative regulation of cell adhesion molecule production (100.0%; 2.547e-10), negative regulation of heterotypic cell-cell adhesion (100.0%; 5.184e-10), regulation of cell-cell adhesion involved in gastrulation (100.0%; 5.184e-10)	7	3	5,06E-13	205,58	205,58

LRG, G-CSF, STAT3, STAT5, JAK1	cellular response to interleukin-6 (50.0%; 8.231e-13), response to interleukin-6 (50.0%; 1.643e-12), interleukin-6-mediated signaling pathway (41.7%; 4.827e-12), receptor signaling pathway via JAK-STAT (41.7%; 6.952e-11), receptor signaling pathway via STAT (41.7%; 7.570e-11)	12	4	1,240E-15	173,35	173,35
S1P1 receptor, S1P3 receptor, APOA1, Pre beta-1 HDL, PERM	protein-lipid complex remodeling (81.8%; 7.797e-23), plasma lipoprotein particle remodeling (81.8%; 7.797e-23), protein-containing complex remodeling (81.8%; 1.758e-22), plasma lipoprotein particle organization (81.8%; 2.006e-21), high-density lipoprotein particle remodeling (72.7%; 2.636e-21)	14	3	1,550E-11	130,01	130,01
Pre beta-1 HDL lipids extracellular region, Pre beta-1 HDL lipids + Cholesterol + Phospholipid = HDL nascent lipids, HDL nascent lipids extracellular region, Cholesterol extracellular region	cholesterol import (100.0%; 2.069e-10), sterol import (100.0%; 2.069e-10), negative regulation of cell adhesion molecule production (100.0%; 2.547e-10), negative regulation of heterotypic cell-cell adhesion (100.0%; 5.184e-10), regulation of cell-cell adhesion involved in gastrulation (100.0%; 5.184e-10)	7	3	2,820E-13	225,21	225,21
APOC3, LPL, Decorin proteoglycan, Biglycan proteoglycan, Versican proteoglycan	chylomicron remodeling (53.8%; 7.041e-18), triglyceride-rich lipoprotein particle remodeling (53.8%; 1.406e-17), lipoprotein metabolic process (69.2%; 3.237e-17), regulation of plasma lipoprotein particle levels (61.5%; 3.097e-16), protein-lipid complex remodeling (53.8%; 5.500e-16)	19	2	2,810E-07	70,75	70,75

Supplementary Table S3: MetaCore Analysis: Comparison of canonical pathways by gene ontology differential proteins in benralizumab (grey) and mepolizumab (black) time points. Red square indicates the canonical pathway involving transthyretin, which was found differential only in benralizumab time points analysis.
