# Supplementary figures and images for: Serum Proteomic Profile of Asthmatic Patients after Six Months of Benralizumab and Mepolizumab Treatment
Source: Biomedicines. 2022 Mar 24;10(4):761. doi: 10.3390/biomedicines10040761 (PMC9027545; doi:10.3390/biomedicines10040761)

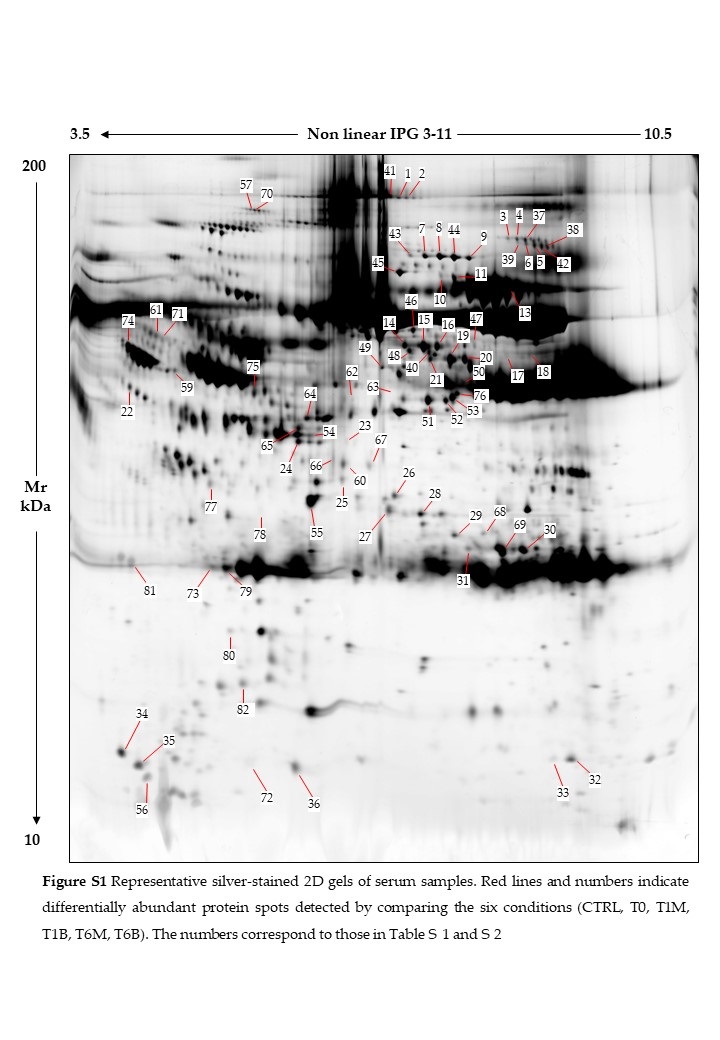

Supplement: Supplementary file 1 [file biomedicines-10-00761-s001.zip › Supplementary Figure S1 Representative silver-stained 2D gels of serum samples.jpg]
